# Sub-surface drip fertigation improves seed cotton yield and monetary returns

**DOI:** 10.3389/fpls.2022.1038163

**Published:** 2022-11-24

**Authors:** Kulvir Singh, Prabhsimran Singh, Manpreet Singh, Sudhir Kumar Mishra, Rashid Iqbal, Ibrahim Al-Ashkar, Muhammad Habib-ur-Rahman, Ayman El Sabagh

**Affiliations:** ^1^ Regional Research Station, Punjab Agricultural University, Faridkot, Punjab, India; ^2^ Punjab Agricultural University, Regional Research Station, Abohar, Punjab, India; ^3^ Department of Agronomy, Faculty of Agriculture and Environment, The Islamia University of Bahawalpur, Bahawalpur, Pakistan; ^4^ Department of Plant Production, College of Food and Agriculture, King Saud University, Riyadh, Saudi Arabia; ^5^ Crop Science, Institute of Crop Science and Resource Conservation (INRES), University of Bonn, Bonn, Germany; ^6^ Department of Agronomy, Faculty of Agriculture, Kafrelsheikh University, Kafrelsheikh, Egypt

**Keywords:** bio-physical water productivity, drip fertigation, economic water productivity, nitrogen use efficiency, surface flood method, water use efficiency

## Abstract

Surface flood (SF) method is used to irrigate cotton in India, which results in huge wastage of water besides leaching of nutrients. This necessitates the adoption of efficient management strategies to save scarce water without compromising the yield. Therefore, a 2-year field investigation was conducted under two climatic regimes (Faridkot and Abohar) to study the effect of sub-surface drip fertigation (SSDF) on seed cotton yield (SCY), water productivity, nitrogen use efficiency (NUE), and economic parameters in comparison with SF and surface drip fertigation (SDF). The field experiment had a total of eight treatments arranged in a randomized complete block design. Three levels of sub-surface drip irrigation [(SSDI); *i.e.*, 60%, 80%, and 100% of crop evapotranspiration (ETc)] and two N fertigation levels [100% recommended dose of nitrogen (RDN; *i.e.*, 112.5 kg N ha^-1^) and 75% RDN] made up six treatments, while SF (Control 1) and SDF at 80% ETc (Control 2), both with 100% of RDN, served as the controls. Among irrigation regimes, the SSDI levels of 80% ETc and 100% ETc recorded 18.7% (3,240 kg ha^-1^) and 21.1% (3,305 kg ha^-1^) higher SCY compared with SF (2,728 kg ha^-1^). Water use efficiency under SF (57.0%) was reduced by 34.2%, 40.8%, and 38.2% compared with SSDI’s 60 (76.5%), 80 (80.3%), and 100% ETc (78.8%), respectively. Among fertigation levels, NUE was higher by 19.2% under 75% (34.1 kg SCY kg^-1^ N) over 100% RDN (28.6 kg SCY kg^-1^ N), but later it also registered 11.9% higher SCY, indicating such to be optimum for better productivity. SSDF at 80% ETc along with 112.5 kg N ha^-1^ recorded 26.6% better SCY (3455 kg ha^-1^) and 18.5% higher NUE (30.7 kg SCY kg^-1^ N) over SF. These findings demonstrate that the application of SSDF could save irrigation water, enhance SCY, and improve the farmers’ returns compared with SF. Therefore, in northwestern India, SSDF at 80% ETc along with 112.5 kg N ha^-1^ could be a novel water-savvy concept which would be immensely helpful in enhancing cotton productivity.

## Introduction

Cotton (*Gossypium hirsutum* L.) is among the most important cash crops being cultivated in India and sustains the nation’s largest organized textile industry. However, India accounts for the greatest area (13.4 m ha) and highest production of cotton in the world (37.1 million bales of 170 kg each), but its mean productivity (487 kg lint ha^-1^) is very low (Anonymous, 2021). More than 65% of Indian cotton is rainfed, with the exception of the northwestern cotton belt (constituted by Punjab, Haryana, and Rajasthan states), where irrigated cotton is cultivated. In Punjab, cotton is mainly grown in the southwestern districts which are characterized by lightly textured soils, brackish groundwater, and arid climate with limited availability of canal water for irrigation (Singh et al., 2018). Here cotton crop is traditionally irrigated through the surface flood (SF) method at four to six growth stages with 75 mm of water required for a single irrigation. Irrigation for agriculture has been the leading consumer of water on the earth, so the gradual decline of water resources is posing a serious concern on the agriculture sector and insisting upon how to operate in a sustainable manner under the ever-growing concern on water scarcity for agrarian usage (Sinha et al., 2017).

This necessitates the adoption of improved and efficient water application strategies to increase the crop productivity with better irrigation management (FernÃ¡ndez et al., 2020; Mishra et al., 2021). To achieve food and fiber security for the ever-increasing population, a globally irrigated agricultural area needs to be increased by 20%, with about 40% increase in irrigated crop yield by year 2025 (Hashem et al., 2018). Different irrigation systems such as surface drip irrigation (SDI), sub-surface drip irrigation (SSDI), and sprinkler irrigation have been found to improve the irrigation efficiency (Ines et al., 2006). Drip fertigation exposes the crop to a certain level of water stress during crop growth stages without sacrificing the crop yield (Pereira et al., 2012) besides enhancing the water use efficiency and uptake of nutrients (Kaur and Brar, 2016; Gondal et al., 2021). In cotton, drip irrigation (DI) resulted in 18%–42% saving of water over furrow irrigation (Ibragimov et al., 2007) and up to 62.1% saving over SF method (Singh et al., 2018).

Nowadays, SSDI is also gaining importance due to more efficient usage of water since there is minimum surface runoff and evaporation from the soil surface as laterals having drippers are buried under the soil surface at regular spacing (Mchugh et al., 2008). SSDI can play a greater role in water management throughout arid and semi-arid regions by applying water and nutrients more precisely to the field in both position and quantity. In SSDI, applied water and nutrients result in higher use efficiency as the topsoil layer is mostly dry and wetting occurs only beneath the soil surface while soil evaporation is controlled (Ben-Gal et al., 2004). SSDI resulted in 20% water saving in organic olive over the conventional method (Martinez and Reca, 2014; Rahim et al., 2020) and 10% higher water use efficiency (WUE) over SDI in cotton (Roopashree et al., 2016). SSDI reduces evaporation from the soil surface compared with SDI and SF because water is delivered directly to the roots as laterals are located within the root zone, thus minimizing water loss. Both SDI and SSDI could be exploited on a large scale especially in northwestern India because this region is constituted by arid and semi-arid areas with a limited supply of irrigation water. Moreover, the groundwater here is brackish and thus unfit for irrigation purposes, which makes this zone ideal for exploiting drip irrigation (Singh et al., 2020).

Fertilizer use efficiency in India can be specifically enhanced greatly over the prevalent but inefficient method of fertilizer application by broadcasting (Harish et al., 2017). Drip-fertigated cotton not only exhibits enhanced N uptake over furrow-irrigated crops but also curbs the energy and labor costs for nutrient application as no extra equipment, labor, and machinery are required (Sidhu et al., 2019). Thus, the SSDF technique can be exploited to realize more crops per drop of water without sacrificing the seed cotton yield SCY besides sustaining cotton productivity. Moreover, fertilizers are applied in split doses under the fertigation system, thus readily facilitating the absorption of nutrients by the crop plants with a minimal problem of nutrient fixation in the soil (Janat, 2008; Imran et al., 2021).

So far, the feasibility of SSDF in cotton agro-ecosystems has been less evaluated in India, and the present study offers an opportunity to fill this gap in the extant agricultural scenario. We hypothesize that SSDF would improve the cotton productivity and also save a huge quantity of water over the conventional practice (surface flood method of irrigation and urea broadcasting) prevalent in India.

Thus, the present study investigated the effects of SSDF at varying irrigation and nitrogen levels on SCY, fiber quality, and water productivity indices so as to evaluate study gaps with the following objectives: (1) to assess the effect of SSDI and N fertigation on the growth, yield parameters, and SCY of *Bt* cotton in comparison with the SDI/SF method, (2) to compare the bio-physical water productivity, water use efficiency, and NUE of *Bt* cotton grown under different irrigation regimes and N levels, and (3) to recommend the most efficient SSDF level to maximize the production of quality seed cotton based on monetary evaluation. The present investigation therefore implements an experimental approach to explore this enunciated research query and produce data-based information for its implementation on a large scale under arid climates. The focus was to optimize the SSDF for increasing the yield and water productivity so as to achieve a higher SCY with the additional saving of water and N fertilizer.

## Materials and methods

### Location and weather

A 2-year field trial was conducted at regional research stations (RRS) of Punjab Agricultural University located at Faridkot and Abohar during the summer seasons (April–November) of 2019 and 2020. These Research Stations are located, 96 km apart, in two distinct agro-climatic locations of the southwestern cotton belt of Punjab. The experimental site of RRS Faridkot lies in agro-climatic zone IV at an altitude of 211 m above mean sea level (AMSL) and latitude of 30°40′ N and longitude of 74°44′ E, whereas the experimental site of RRS Abohar lies in agro-climatic zone V at an altitude of 186 m AMSL and latitude of 30°08′ N and longitude of 74°12′ E. Both of the experimental locations fall in the Trans Gangetic plain zone of India with the climate characterized by sub-tropical and semi-arid conditions experiencing a dry and hot summer from mid-April to June and a cold winter season from November to January, thus truly representing the cotton belt of northwestern India. The average annual rainfall of both sites varies from 300 to 400 mm, with 75% of the total precipitation mainly occurring from July to September.

### Design of the experiment

**Figure 1:**
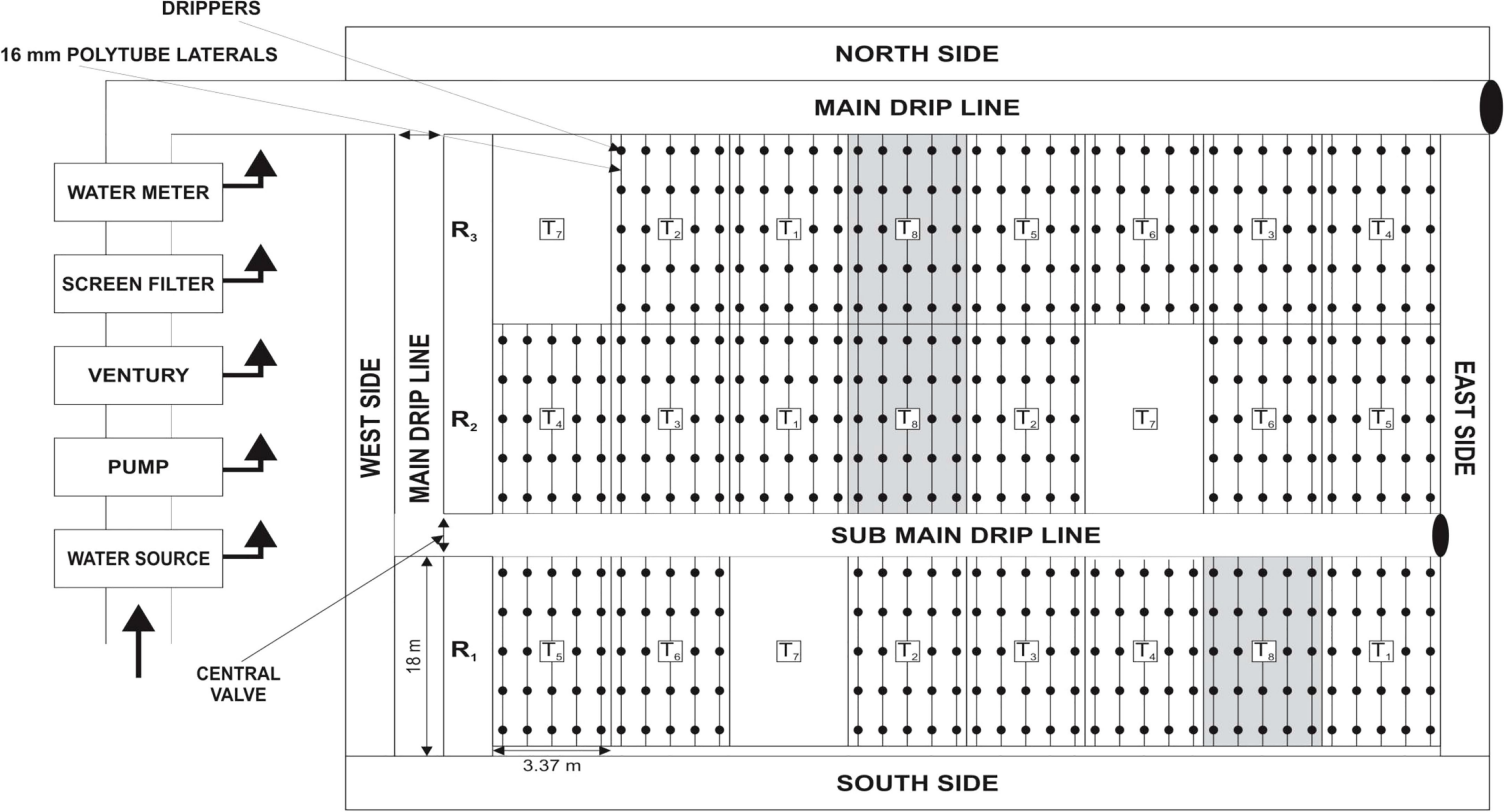
Layout of the experimental plots showing different treatments and drip line placement.

The experiment having eight treatments has been laid out in a randomized complete block design with three replications (**Figure 1**). It was comprised of six treatment combinations from three levels of SSDI [60% crop evapotranspiration (ETc), 80% ETc, and 100% ETc of PAN evapotranspiration] and two N fertigation levels [75% RDN (84.4 kg N ha-1) and 100% RDN (112.5 kg N ha-1)]. In addition, there were two extra absolute Control treatments, *i.e*., SF method of irrigation with 100% RDN through broadcasting of urea (Control 1) and SDI at 0.8 ETc coupled with 112.5 kg N ha-1 (Control 2).

Various treatment combinations included the following: T₁—SSDI at 60% ETc with DF of 75% RDN, T₂—SSDI at 60% ETc with DF of 100% RDN; T₃—SSDI at 80% ETc with DF of 75% RDN; T₄—SSDI at 80% ETc with DF of 100% RDN; T₅—SSDI at 100% ETc with DF of 75% RDN; T₆—SSDI at 100% ETc with DF of 100% RDN; and T₇—SF irrigation with 100% RDN through broadcasting of urea–Control 1; and T_8_—SDI at 80% ETc with DF of 100% RDN (Control 2).

### Methodology and crop management

After harvesting of the wheat crop, the field was ploughed twice, and pre-sowing irrigation (*rauni*) was applied at both study locations. *Bt* cotton hybrid cultivar (RCH773 BGII) was sown in the first fortnight of May during both years (2018 and 2019) at Faridkot and Abohar. The sowing was done manually by hand dibbling method using two seeds per hill at a uniform depth of 5 cm. An inter-row spacing of 67.5 cm and intra-row spacing of 75 cm within plants was uniformly maintained. Each experimental plot (60.75 m^2^) had a total of 125 plants in five rows, while each row length was 18 m.

The sub-surface laterals for SSDI were installed using a tractor-operated machine at a uniform depth of 20 cm, having an inter-row spacing of 67.5 cm with the emitter discharging 2.2 L of water per hour. However, for Control 2, SDI drip laterals with a similar discharge were manually laid out on the ground surface. The Control 1 plots receiving irrigation through SF were delivered with water through dedicated PVC pipes. All the experimental plots received regulated and measured water supply through a water meter and were surrounded by strong bunding all around, with a sufficient buffer area (2.0 m) so that variation owing to water application among different treatments could be minimized. The bulk density of soil has been measured by using the Core method (Black and Hartage, 1986), while soil moisture was determined by employing time domain refractometry (PR2/6 Profile Probe, Delta-T Devices Ltd. UK) after calibrating it with gravimetric methods (Richards and Weaver, 1943).

### Growth- and yield-attributing characteristics and seed cotton yield

Growth- and yield-attributing parameters like plant height, sympodial branches per plant, bolls per plant, and boll weight have been recorded from 10 randomly selected plants per plot. Biomass accumulation was measured by cutting the whole aboveground portion of the plant after harvest and then weighing after thorough drying under the sun. SCY constituted the total weight of both the hand pickings and is expressed in kilograms per hectare (Mishra et al., 2021).

### Irrigation, fertilizer application, and productivity indices

Irrigation under the SDI and SSDI method was applied at an interval of every 5 days. The total amount of applied irrigation water varied according to treatments, which were based on the amount of crop evapo-transpiration. In the SF method (conventional), water was applied by flood irrigation. A total of six and four SF irrigations were applied at the Faridkot and Abohar locations, respectively, during 2019, and the corresponding values were four and five during 2020. In SF plots, the first irrigation was given at 35 days after sowing (DAS) in mid-June at all locations and study years. The second, third, fourth, and fifth (last) SF irrigation, respectively, was applied during the end June, July, August, and September, respectively. The quantity of each irrigation was measured by using a water meter in all the treatment plots, and water applied under all irrigations was summed up to calculate the total irrigation water.

To work out the ETc, reference evapotranspiration (ETo) was calculated with a calculator developed by FAO from site-specific weather data for both locations. According to the user manual given by FAO, to calculate the daily crop evapotranspiration (ETc), the value of the crop coefficient (K_c_) of cotton crop was 0.75 until the end of June, 1.15 during July and August, and 0.70 until boll picking. Fertilizer N in the form of urea (46% N) was applied for SDF and SSDF treatments starting from 35 DAS and delivered in 10 equal splits after every 5 days except for the SF, where 100% RDN was applied in two equal splits, *i.e*., half dose at thinning and the remaining half at flowering. The application of phosphorous was skipped as the recommended dose of P was applied to the preceding wheat as recommended by Punjab Agricultural University (Anonymous, 2022). Actual crop evapotranspiration (ET_a_) has been worked out with the help of the soil water balance equation (FernÃ¡ndez et al., 2020):


(1) $$ET_{a}=\;IW\;+\;P\;–\;D\;–\;R\;\pm \;\Delta S\;$$

where IW is amount of irrigation water applied (mm), P represents precipitation in mm, R indicates surface runoff (mm), D is deep drainage (mm), and ΔS represents soil profile moisture change (mm). Runoff was nil as ridges/buffers surrounded all the plots. Deep drainage was assumed to be zero when moisture storage in the soil profile was lesser than the field capacity and when soil moisture storage (SMS) surpassed the field capacity storage either after a rain or due to irrigation. Thereafter, deep drainage has been developed as the gap between field capacity storage and SMS plus rain/irrigation. Since the water table at both study sites was below 3 m, an upward flux from the groundwater was not considered. Two-meter wide buffers were established between various plots to eliminate water fluxes in the vicinity of root zone laterals.

Bio-physical water productivity (BWP) and economic water productivity (EWP) were computed by using the following equations (Perry et al., 2017; FernÃ¡ndez et al., 2020):


(2) $$BWP\text{ (}kg\;m^{-3}\text{) }=\;SCY/ET_{a}$$

(3) $$EWP\text{ (}\$\;m^{-3}\text{) }=\;NR/ET_{a}$$

where SCY means seed cotton yield (kg ha^-1^), ET_a_ is actual crop evapotranspiration (m^3^ ha^-1^), and NR indicates net returns ($ ha^-1^).

WUE was calculated by using the following equations (Perry et al., 2009; Sahoo et al., 2018):


(4) $$WUE\;=\;ET_{a}/IW\;+\;R\;\pm \;\Delta S\;$$

where IW is irrigation water applied (m^3^ ha^-1^), R represents rainfall (m^3^ ha^-1^), and ±ΔS was change in soil profile moisture (m^3^ ha^-1^), while ETa indicates actual crop evapotranspiration (m^3^ ha^-1^).

Nitrogen use efficiency (NUE) is calculated using the formula given below:


(5) NUE = seed cotton yield/N applied 

### Fiber quality parameters

Lint samples were obtained by ginning the clean and dried weighed samples of seed cotton through a single-roller electric gin, and ginning turnout (GTO) was computed by using the following formula:


(6) GTO (%) = (weight of lint in grams/weight of seed cotton in grams) × 100 

Fiber samples were sent to the laboratory of ICAR-Central Institute for Research on Cotton Technology (ICAR-CIRCOT), Mumbai, for measurement of various fiber parameters like halo length, fiber strength, uniformity index, micronaire, *etc*. A sample of 100-gram lint was taken to measure the micronaire value by using Prectitronic Digital Mic Tester at C, Mumbai.

### Monetary evaluation

The total cost of cultivation incurred for raising cotton crop was calculated with the help of Enterprise budget (2021) of *Kharif* crops by the Department of Economics and Sociology, PAU, Ludhiana. Gross returns of different treatments were worked out by multiplying the SCY from respective treatments with the prevalent market price of $0.75 kg^-1^ of seed cotton. The benefit/cost ratio (B:C) was worked out to check the economic feasibility of treatments and is calculated by dividing the net returns by the total cost of cultivation (Singh et al., 2020).

### Statistical analysis

Statistical analysis of various recorded parameters and calculated indices was performed to evaluate the effect of various treatments (SSDI regimes and N fertigation levels), in comparison with control treatments, using SAS Proc GLM (SAS software 9.3, SAS Institute Ltd., USA). Significant mean differences were compared using Fisher’s least significant difference test at a probability level of 5%. The variance of data in both sites was homogeneous according to Bartlett’s test (*p* ≤ 0.05), so the data of both sites and years was pooled and analyzed.

## Results and discussion

### Weather and site characteristics

**Figure 2:**
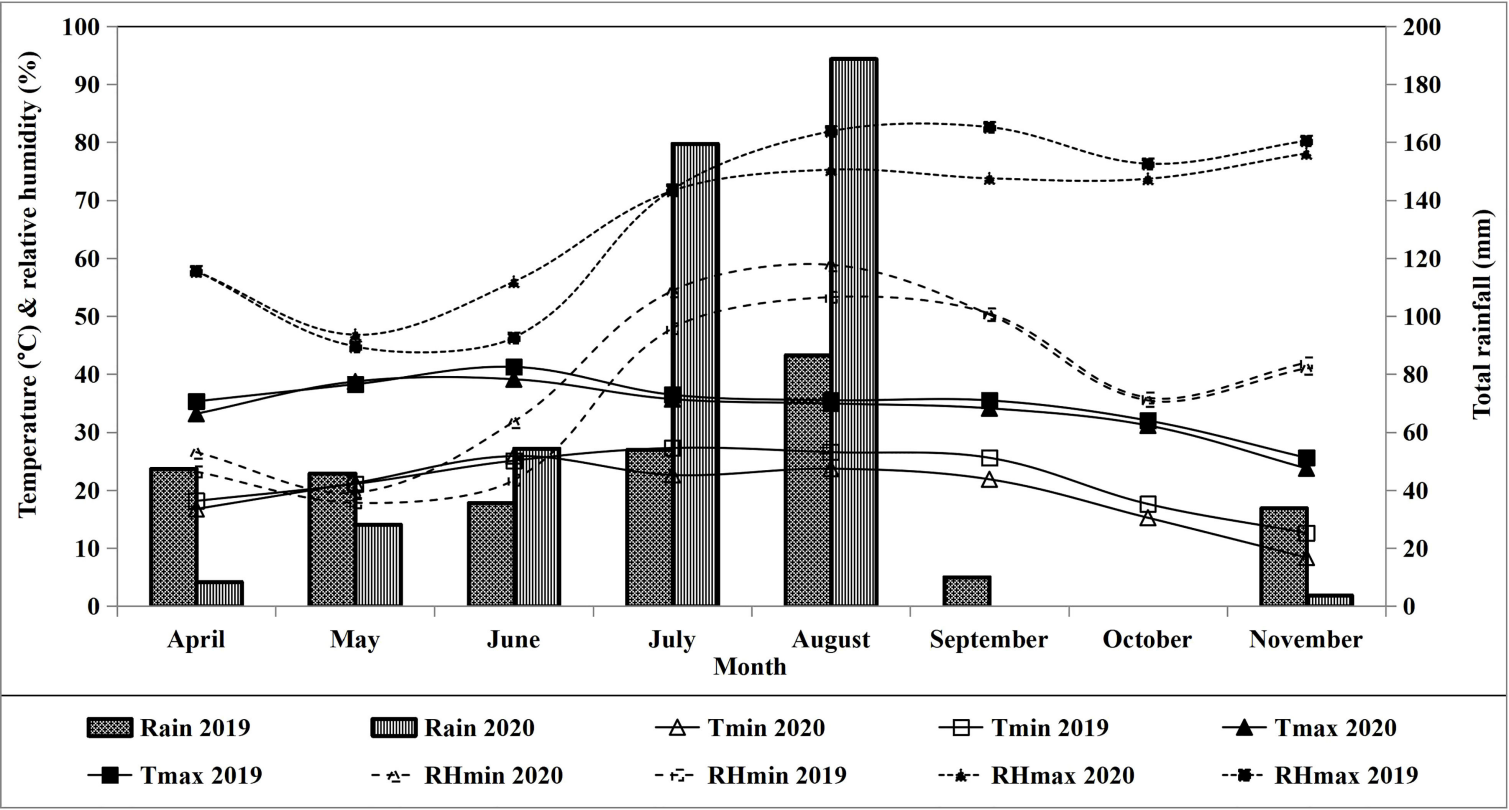
Weather data of the experimental site at Abohar during crop growth periods 2019 and 2020. RHm, maximum relative humidity; RHe, minimum relative humidity; Tmax, maximum temperature; Tmin, minimum temperature.

**Figure 3:**
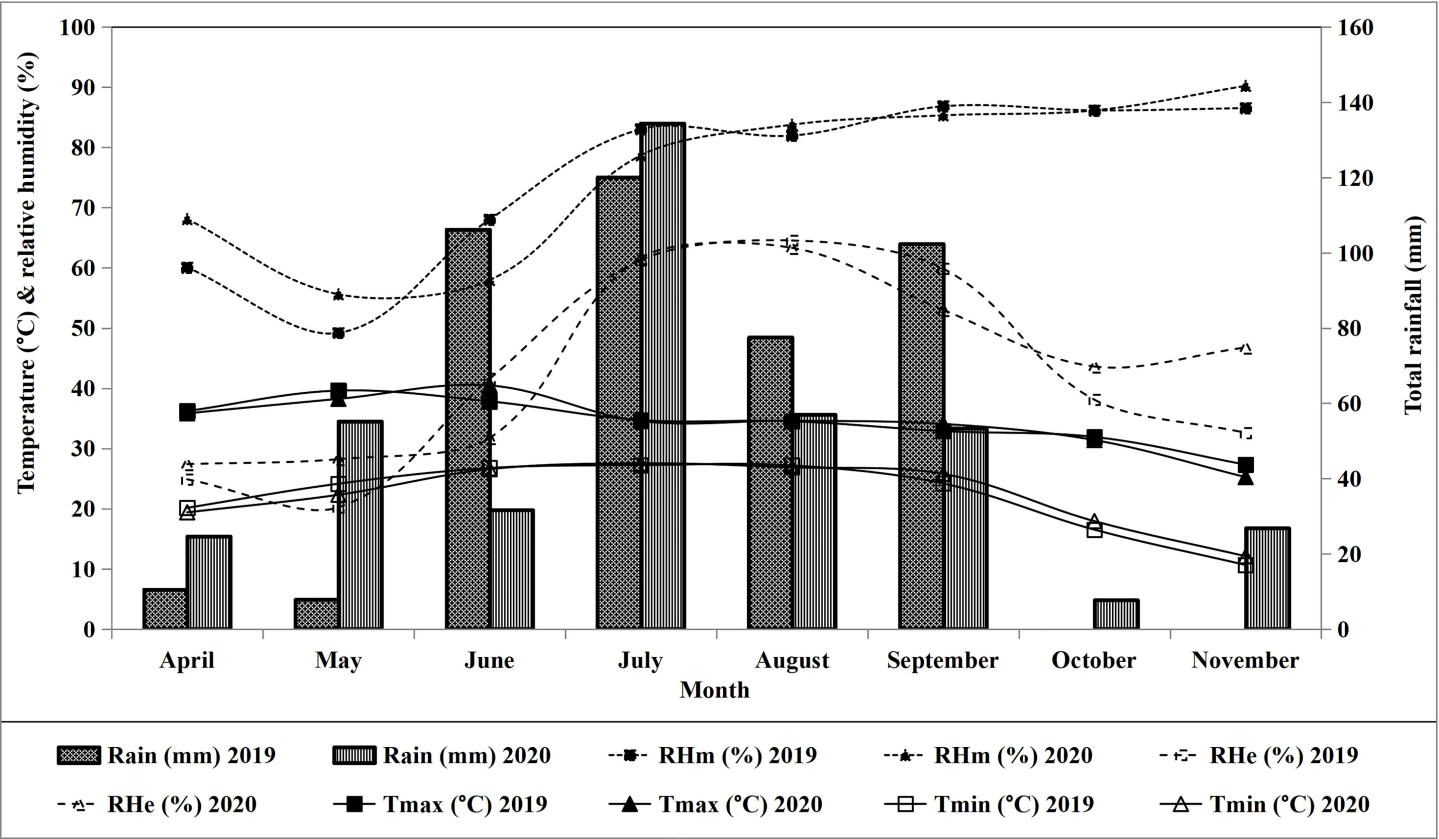
Weather data of the experimental site at Faridkot during crop growth periods 2019 and 2020. RHm, maximum relative humidity; RHe, minimum relative humidity; Tmax, maximum temperature; Tmin, minimum temperature.

The data pertaining to weather-related parameters has been recorded from the agro-meteorological observatories of RRS, Faridkot, and RRS, Abohar and presented in **Figures 2**, **3**.

Among the different treatments, the actual crop evapotranspiration (ET_a_) ranged from 345.8 to 588.8 mm at both locations over the years. During 2019, except for the SF, negative soil moisture confirmed the necessity of underground water withdrawal for meeting irrigation demand that was substantially improved in the later year for each treatment at both experimental sites. The effects of the available moisture in the soil profile witnessed the heavy deep drainage during 2020 compared with the year 2019. Similarly, a huge surface runoff, *i.e.*, 86.4 and 88.2 mm at Faridkot and 92.3 and 93.3 mm at Abohar, was recorded for the surface flood method which was tremendously reduced under drip treatments. The data on the physio-chemical characteristics of soil at the experimental sites is given in **Table 1**. The layer-wise moisture retention capacity (at field capacity and permanent wilting point, PWP) of soil at both experimental sites for soil profile (0–150 cm) has been worked out and presented to work out various water indices (**Table 2**). Layer wise bulk density of both experimental sites is presented in **Table 3**. The detailed information on cultivar, planting geometry, sowing and harvesting dates, *etc*., is summarized (**Table 4**).

**Table 1:** Chemical properties of soil at the experimental sites.

Soil properties	Faridkot		Abohar		Analytical method used
	Value	Rating	Value	Rating	
pH	8.3	Normal	8.3	Normal	Beckman’s glass electrode pH meter (Jackson, 1967)
EC (dS m^-1^)	0.16	Normal	0.18	Normal	Solubridge conductivity meter (Jackson, 1967)
Organic carbon (%)	0.51	Medium	0.42	Medium	Rapid titration method (Walkley and Black, 1934)
Available nitrogen (kg ha^-1^)	188	Medium	192	Medium	Alkaline potassium permanganate method (Subbiah and Asija, 1956)
Available phosphorus (kg ha^-1^)	21.2	Medium	35	High	0.5 M sodium bicarbonate extractable P method (Olsen et al., 1954)
Available potassium (kg ha^-1^)	638	High	530	High	Ammonium acetate extractable K method (Merwin and Peech, 1950)
Soil texture	Sandy loam		Sandy loam		International pipette method (Piper, 1966)

**Table 2:** Layer-wise moisture retention capacity of soil at the experimental sites.

Soil depth (cm)	Volumetric moisture content (%) at field capacity		Volumetric moisture content (%) at permanent wilting point	
	Faridkot	Abohar	Faridkot	Abohar
0–10	21.2	19.9	12.3	8.6
11–20	16.9	14.5	9.9	9.9
20–30	16.1	15.1	9.6	10.1
30–40	18.0	16.2	10.7	9.8
40–60	17.8	16.5	10.2	8.1
60–100	16.1	14.9	9.8	9.0

**Table 3:** Layer wise bulk density of experimental sites.

Soil depth (cm)	Bulk density (g cm^-3^)	
	Faridkot	Abohar
0–15	1.56	1.58
15–30	1.54	1.68
30–60	1.66	1.72
60–90	1.61	1.59
90–120	1.60	1.63
120–150	1.58	1.65

**Table 4:** Details of crop, cultivars, planting geometry, and sowing and harvesting dates.

Crop	Cultivars	Planting geometry(cm)	Date of sowing		Date of harvesting			
			RRSA	RRSF	RRSA		RRSF		
Cotton	RCH 773 BGII RCH 773 BGII	67.5 × 75 67.5 × 75	23.05.2019	09.05.2019	21.10.2019 (first picking)	6.11.2019 (second picking)	18.10.2019 (first picking)	6.11.2019 (second picking)	
			05.05.2020	16.05.2020	17.10.2020 (first picking)	4.11.2020 (second picking)	19.10.2020 (first picking)	5.11.2020 (second picking)	

A, RRSA (Abohar); F, RRSF (Faridkot).

### Growth parameters and biomass accumulation by cotton

Different irrigation and fertigation treatments exerted a significant effect on growth parameters like plant height and biomass accumulation (**Table 5**). Among the tested irrigation regimes, taller plants were recorded under SDI (170.0 cm), closely followed by a SSDI level of 80% ETc (164.3 cm) and 100% ETc (167.4 cm), while a significant reduction in plant height was observed under 60% ETc (148.1 cm) and SF (156.1 cm). Biomass accumulation has been highest under a SSDI level of 100% ETc (2,627 g m^-2^) compared with 80% ETc (2439 g m^-2^) and 60% ETc (1978 g m^-2^). The reduced plant height and biomass accumulation under SSDI level of 60% ETc might be due to the fact that here least water was supplied, which, in turn, failed to maintain optimal crop growth. The better plant vigor under SDI over SF in the present findings is in close proximity with those of Yadav and Chauhan (2016), who observed taller plants under SDI over SF. Furthermore, SF not only recorded shorter plants in line with Prajapati and Subbaiah (2018) but also lesser biomass accumulation (2,138 g m^-2^) in comparison with various SDF/SSDF treatments under 80%/100% ETc, elucidating that applied water and nitrogen could not be efficiently utilized (**Table 5**).

**Table 5:** Effect of various treatments on growth parameters, yield attributes, and seed cotton yield.

Nitrogen fertigation schedules (FS)	Irrigation regimes (IR)					
	60% ETc	80% ETc	100% ETc	Mean	Control 1	Control 2
Plant height (cm)						
75% RDN	144.4	162.2	166.1	157.6	156.1	170
100% RDN	151.9	166.3	168.8	162.3		
Mean	148.1	164.3	167.4			
LSD (p=0.05)	IR = 4.6 ; FS = 3.8; IR*FS = NS; IR*FS vs. Controls = 5.2					
Sympodial branches plant^-1^						
75% RDN	16.8	21	22.2	20	20.5	24.2
100% RDN	18.4	23.3	24	21.9		
Mean	17.6	22.2	23.1			
LSD (p=0.05)	IR = 0.95 ; FS = 0.77; IR*FS =NS; IR*FS vs. Controls =1.10					
Bolls plant^-1^						
75% RDN	44.5	54.1	58	52.2	46.5	56.9
100% RDN	48.9	60.4	62.5	57.2		
Mean	46.7	57.2	60.2			
LSD (p=0.05)	IR = 1.9 ; FS =1.5 ; IR*FS = NS; IR*FS vs. Controls =2.3					
Boll weight (g)						
75% RDN	3.58	3.9	4.01	3.83	3.81	4.11
100% RDN	3.7	4.01	4.07	3.92		
Mean	3.64	3.95	4.04			
LSD (p=0.05)	IR =0.11 ; FS =0.09; IR*FS = NS; IR*FS vs. Controls = 0.13					
Seed cotton yield (kg ha^-1^)						
75% RDN	2490	3024	3133	2882	2728	3300
100% RDN	2747	3455	3477	3226		
Mean	2619	3240	3305			
LSD (p=0.05)	IR = 120; FS = 98 ; IR*FS = NS; IR*FS vs. Controls = 143					
Biomass accumulation (g m^-2^)						
75% RDN	1867	2361	2601	2276	2138	2433
100% RDN	2090	2518	2652	2420		
Mean	1978	2439	2627			
LSD (p=0.05)	IR = 62 ; FS =50; IR*FS =NS; IR*FS vs. Controls = 83					

Among N fertigation schedules, higher plant height and biomass accumulation was evident under 100% RDN (2,420 g m^-2^) over the 75% RDN (2276 g m^-2^). Nevertheless, both SSDF levels of 75% RDN and 100% RDN revealed better plant height and biomass accumulation over the SF method (2,138 g m^-2^), which could be attributed to the optimum availability of water and nitrogen to plants under SSDF, in agreement with Ayyadurai and Manickasundaram (2014) who recorded 36% higher biomass under DF of 100% RDN over soil application under SF. These findings established water and nitrogen to be among the essential growth factors as evident from better height and biomass accumulation under their increased supply (Brar et al., 2021). Higher biomass accumulation under SSDI over SF method is also supported by Sampthkumar et al. (2006) who recorded improved biomass accumulation under DI over border strip and SF irrigation.

### Yield attributes and seed cotton yield

Sympodial branches, bolls per plant, boll weight, and SCY varied significantly under various irrigation regimes (**Table 5**). Higher sympodial branches per plant were recorded under SDI (24.2), closely followed by SSDI of 100% ETc (23.1), 80% ETc (22.2), and SF method (20.5), while the number was least under SSDI of 60% ETc (17.6). The observation on improved sympodial branches per plant under SSDI is in conformity with that of Ali et al. (2017) who reported 15% higher sympodial branches under SDI over the furrow method. Higher bolls per plant was revealed under a SSDI of 100% ETc (60.2) and 80% ETc (57.2) over SSDI of 60% ETc (46.7) and SF method (46.5), which was in agreement with Prajapati and Subbaiah (2018) who recorded improved bolls per plant under SDI over the furrow method. A significant reduction by 23% and 29% for bolls per plant under the SF method over a SSDI of 80% ETc and 100% ETc was evident. Higher boll weight under SDI (4.11 g) was closely followed by SSDI at 100% ETc (4.04 g) and 80% ETc (3.95 g), while SSDI at 60% ETc recorded the significantly lowest value (3.64 g) primarily due to reduced water supply (Singh et al., 2018).

Among SSDI levels, SCY was maximum at 100% ETc (3,305 kg ha^-1^), closely followed by 80% ETc (3,240 kg ha^-1^) and with the least value under 60% ETc (2619 kg ha^-1^), where it was 26% and 23.7% lower compared with 80% ETc and 100% ETc, respectively. Nevertheless, SDI recorded statistically at par SCY (3,300 kg ha^-1^) with SSDI of 80% ETc and 100% ETc. However, SSDI of 80% and 100% ETc resulted in 18.7% and 21% higher SCY over SF (2,728 kg ha^-1^) due to better yield parameters (Aladakatti et al., 2012). This was primarily due to the improved boll count per plant which was 23% and 29.4% higher under SSDI level of 80% ETc and 100% ETc, respectively, over the SF method. Singh et al. (2018) also reported higher SCY by 19% and 23% over the conventional SF method under SDI of 100% ETc and 80% ETc, owing to the improved boll count. Better SCY and yield attributes such as higher bolls per plant under SSDI of 100% ETc and 80% ETc over SF irrigation are also supported by Neelakanth et al. (2019).

A fertigation level of 100% RDN elucidated better sympodial branches per plant (21.9), boll weight (3.92 g), and bolls per plant (57.2) over 75% RDN (20.0, 3.83 g, and 52.2, respectively) in conformity with Singh and Bhati (2018) who observed higher bolls per plant under SDI of 100% RDN (50.8) over the 75% RDN (46.2). Among N levels, 100% RDN revealed significantly higher SCY by 11.9% and 18.2% over the 75% RDN (2,882 kg ha^-1^) and broadcasting method (2,728 kg ha^-1^), respectively. Furthermore, the data elucidated that SSDF of 100% RDN either at 80% (3,455 kg ha^-1^) or 100% ETc (3,477 kg ha^-1^) improved SCY by 4.6% and 5.3%, respectively, over SDF.

**Figure 4:**
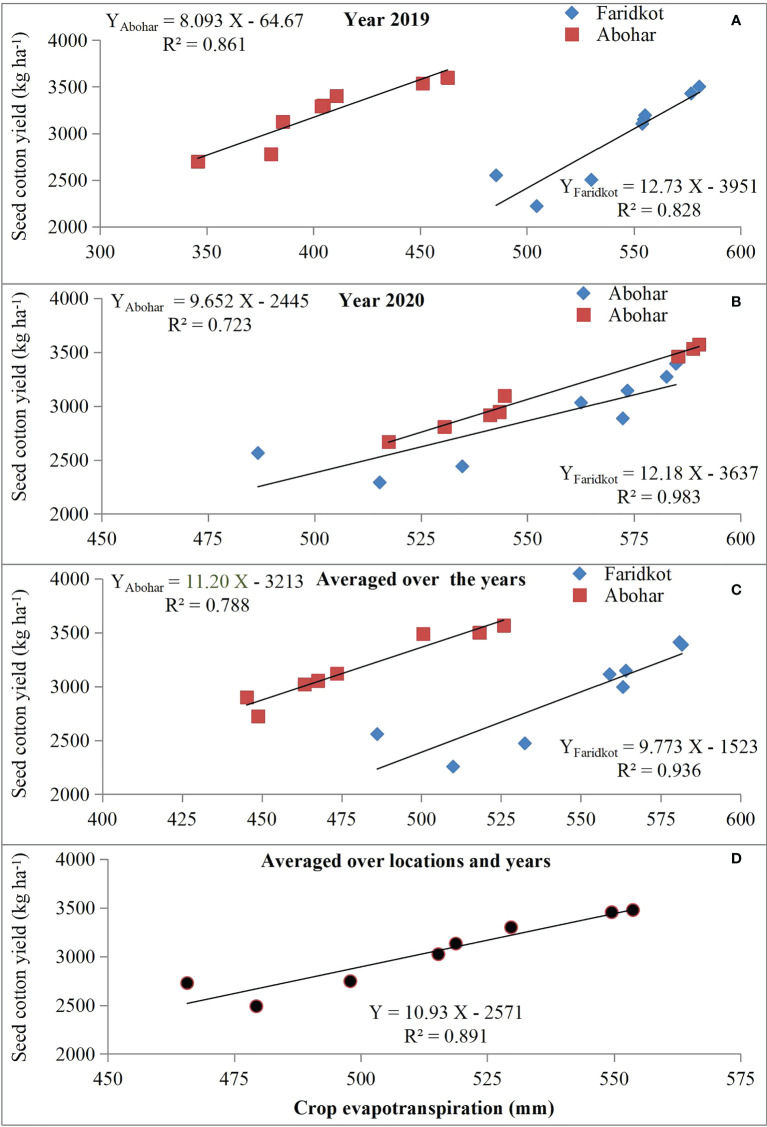
Seed cotton yield response to actual crop evapotranspiration during 2019 **(A)** and 2020 **(B)**, averaged over the years for each location **(C)** and averaged over the locations and years **(D)**.

**Figure 5:**
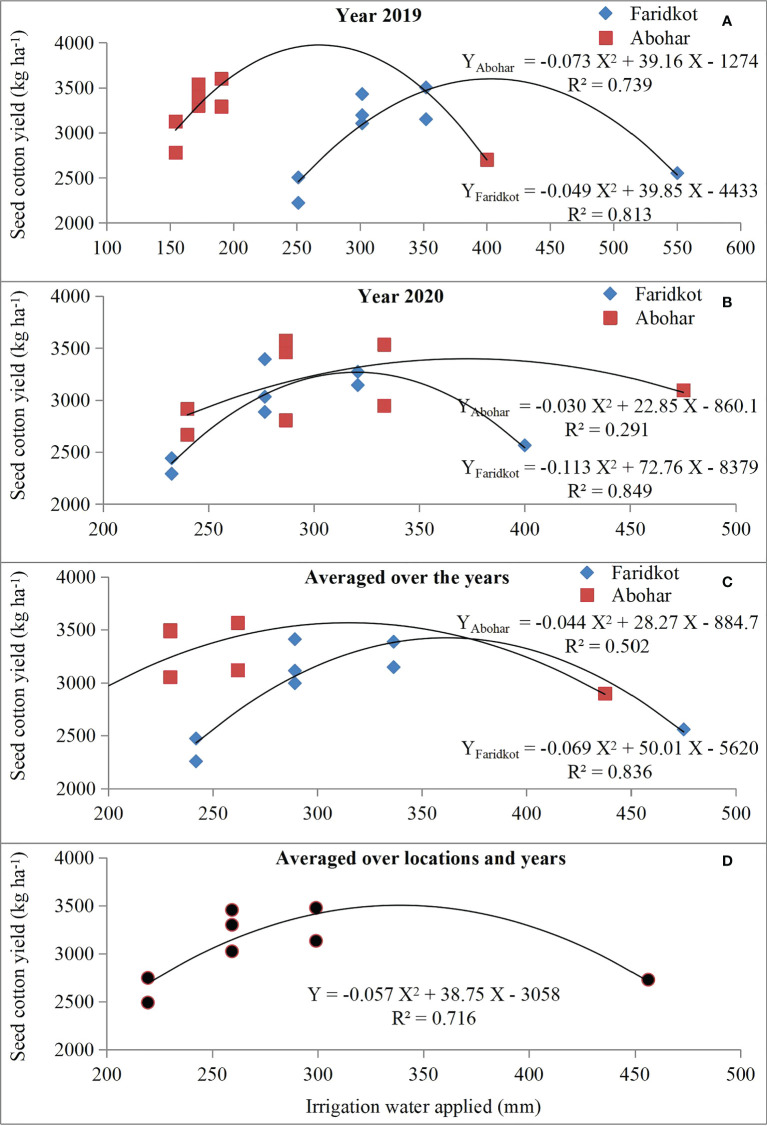
Seed cotton yield response to irrigation water applied (mm) during 2019 **(A)** and 2020 **(B)**, averaged over the years for each location **(C)** and averaged over the locations and years **(D)**.

**Figure 6:**
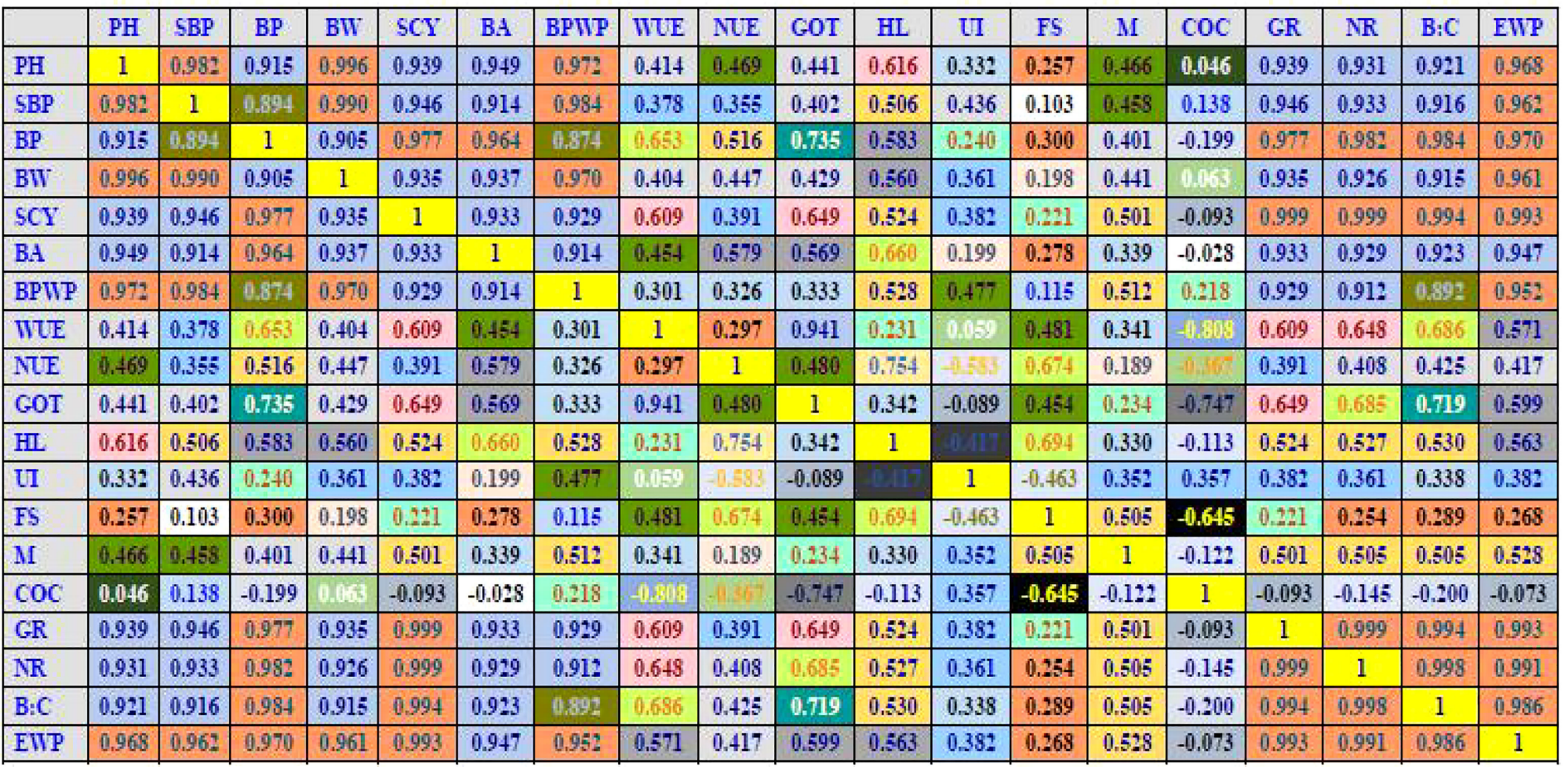
Correlation heat map among various traits of cotton. PH, plant height (cm); SBP, sympodial branches plant⁻¹; BP, Bolls per plant; BW, Boll weight (g); SCY, seed cotton yield (kg ha⁻¹); BA, biomass accumulation (g m⁻²); BPWP, bio-physical water productivity (kg m⁻³); WUE, water use efficiency (%); NUE, nitrogen use efficiency (kg SCY kg⁻¹ N); GOT, Ginning turnout (%); HL, halo length (mm); UI, uniformity index; FS, fiber strength (g tex⁻¹); M, micronaire; COC, cost of cultivation ($ ha⁻¹); GR, gross returns ($ ha⁻¹); NR, net returns ($ ha⁻¹); B:C, benefit/cost ratio; EWP, economic water productivity ($ m⁻³).

Seed cotton yield revealed a positive and linear relationship (**Figure 4**) with crop evapotranspiration (ET_a_), while its relationship with irrigation water applied (IWA) followed a second-order polynomial trend (**Figure 5**). Furthermore, the *R* ^2^ values for IWA and SCY for 2019 (**Figure 5A**), 2020 (**Figure 5B**), averaged over the years (**Figure 5C**), and averaged over the locations and years were significant (**Figure 5D**). The regression equations for individual year and location and also when averaged over the years and locations clearly elucidate SCY to be dependent upon ET_a_ (**Figures 4A–D**) and IWA (**Figures 5A–D**). A correlation heat map among various traits of cotton is given in **Figure 6**. This further signifies that efficient usage of water and nutrients can be made under SSDF as evident from better yield realization. These results clearly established that SSDF could play a pivotal role in improving the yield attributes and SCY compared with SF and the soil application of nutrients. Nevertheless, improved boll weight and bolls per plant under SSDF have remained as the primary reasons for the higher SCY. The boll count per plant was 12.2% and 23.0% higher under the SSDF of 75% RDN and 100% RDN, respectively, over broadcasting of 100% RDN in SF. These findings reveal that both water and nitrogen greatly govern the yield as envisaged from the fact that, by increasing the water and N fertigation level, crop yield tends to improve (Dar et al., 2017; Thanappan et al., 2020).

A significant reduction in yield attributes and SCY is evident under the least level of water and nitrogen (*i*.*e*., SSDI of 60% ETc along with fertigation of 75% RDN), where the supply of both water and nitrogen was minimal (**Table 5**). SCY improved significantly by 23.7%, while the increasing irrigation level from 60% ETc to 80% ETc was in conformity with Singh et al. (2018). However, there was only a marginal increase of 2% while moving from 80% ETc to 100% ETc. The present data manifested that, under 100% RDN, SSDI—at either 80% ETc or 100% ETc—remained on par for SCY, though it increased by 708 and 730 kg ha^-1^, respectively, over the SSDF at 60% ETc. This revealed that SCY exhibited a tendency to increase between the irrigation levels of 80% ETc and 100% ETc but was optimized at 80% ETc (Kumar et al., 2021).

### Effect of different treatments on water productivity indices

The total irrigation water applied has been highest under SF (Control 1), *i*.*e*., 550 and 400 mm during 2019, with a value of 400 and 475 mm during 2020 for Faridkot and Abohar, respectively (**Tables 6**, **7**). However, actual crop evapotranspiration (ET_a_) remained higher for SSDI at 100% ETc with 100% RDN (580.6 and 462.8 mm for 2019; 582.6 and 588.8 mm for 2020) for Faridkot and Abohar, respectively. A general reduction for ET_a_ was observed under SF (Control 1) compared with SSDI treatments across the locations. The ETa followed a linear relationship with SCY among the studied treatments over the locations during both study years (**Figures 4A–D**). During 2019, ETa was highest under T_6_ (*i*.*e*., 580.6 and 462.8 mm for Faridkot and Abohar, respectively), while during 2020, T_4_ (584.8_ mm_) and T_8_ (590.2 mm) revealed higher ETa values for Faridkot and Abohar, respectively. These findings clearly elucidated a saving of 36–54.3% and 52.4-61.4% of irrigation water under SSDI for Faridkot and Abohar, respectively, during 2019 with increased ET_a_ over SF (Cetin and Kara, 2019). During 2020, the corresponding values ranged from 19.8–41.8% to 30.0–50.5%. The presented results on savings of water under SSDI are well supported by Patil et al. (2009) who observed 45.6% water saving under DI at 100% ETc over the SF method. Roopashree et al. (2016) also reported a saving of 21.7% irrigation water under SSDI over SF.

**Table 6:** Irrigation water applied and actual crop evapotranspiration under various treatments (2019).

	Faridkot						Abohar					
	IWA (mm)	RF (mm)	Δ S (mm)	ET_a_ (mm)	D (mm)	R. (mm)	IWA (mm)	RF (mm)	Δ S (mm)	ET_a_ (mm)	D (mm)	R. (mm)
T_1_: SSDI at 60% crop evapotranspiration (ETc) with DF of 75% RDN	232.5	486.3	38.3	515.3	114.6	50.6	239.9	457.8	33.3	517.4	94.3	52.7
T_2_: SSDI at 60% ETc with DF of 100% RDN	232.5	486.3	34.8	534.7	102.1	47.2	239.9	457.8	22.5	541.2	83.4	50.6
T_3_: SSDI at 80% ETc with DF of 75% RDN	276.7	486.3	40.7	572.3	109.9	40.1	286.6	457.8	58.7	530.5	99.6	55.6
T_4_: SSDI at 80% ETc with DF of 100% RDN	276.7	486.3	35.9	584.8	97.5	44.8	286.6	457.8	26.2	585.3	84.6	48.3
T_5_: SSDI at 100% ETc with DF of 75% RDN	320.8	486.3	51.5	573.4	129.4	52.8	333.2	457.8	79.1	543.4	110.1	58.4
T_6_: SSDI at 100% ETc with DF of 100% RDN	320.8	486.3	48.6	582.6	125.8	50.1	333.2	457.8	68.2	588.8	86.7	47.3
T_7_: surface flood with 100% RDN (Control 1)	400.0	486.3	62.8	486.7	248.6	88.2	475.0	457.8	95.8	544.6	200.1	92.3
T_8_: surface drip at 80% ETc with 100% RDN (Control 2)	276.7	486.3	58.7	562.6	90.1	51.6	286.6	457.8	39.7	590.2	68.3	46.2

IWA, irrigation water applied; RF, rainfall; ΔS, change in soil profile moisture; ET_a_, actual crop evapotranspiration; D, drainage; R, runoff; SSDI, subsurface drip irrigation; DF, drip fertigation; RDN, recommended dose of nitrogen.

**Table 7:** Irrigation water applied and actual crop evapotranspiration under various treatments (2020).

Treatment	Faridkot						Abohar					
	IWA (mm)	RF (mm)	ΔS (mm)	ET_a_ (mm)	D (mm)	R. (mm)	IWA (mm)	RF (mm)	ΔS (mm)	ET_a_ (mm)	D (mm)	R. (mm)
T_1_: SSDI at 60% crop evapotranspiration (ETc) with DF of 75% RDN	251.2	366.1	-21.0	504.6	94.3	39.4	154.3	353.3	-18.2	380.1	89.6	56.1
T_2_: SSDI at 60% ETc with DF of 100% RDN	251.2	366.1	-19.4	530.2	72.5	34.0	154.3	353.3	-16.5	385.6	92.3	46.2
T_3_: SSDI at 80% ETc with DF of 75% RDN	301.6	366.1	-17.2	553.9	99.0	32.0	172.3	353.3	-12.8	404.6	98.2	35.6
T_4_: SSDI at 80% ETc with DF of 100% RDN	301.6	366.1	-18.1	576.8	74.6	34.4	172.3	353.3	-10.1	451.1	69.2	15.4
T_5_: SSDI at 100% ETc with DF of 75% RDN	352.0	366.1	-10.9	554.6	139.3	35.1	190.4	353.3	-4.8	403.7	99.2	45.6
T_6_: SSDI at 100% ETc with DF of 100% RDN	352.0	366.1	-8.5	580.6	109.4	36.4	190.4	353.3	-2.1	462.8	70.4	12.6
T_7_: surface flood with 100% RDN (Control 1)	550.0	366.1	45.0	485.6	299.1	86.4	400.0	353.3	32.8	345.8	281.4	93.3
T_8_: surface drip at 80% ETc with 100% RDN (Control 2)	301.6	366.1	-6.7	555.2	73.9	45.3	172.3	353.3	-4.3	410.8	80.2	38.9

IWA, irrigation water applied; RF, rainfall; ΔS, change in soil profile moisture; ET_a_, actual crop evapotranspiration; D, drainage; R, runoff; SSDI, subsurface drip irrigation; DF, drip fertigation; RDN, recommended dose of nitrogen.

Among the studied irrigation regimes, improved BWP values (**Table 8**) were recorded under SSDI levels of 100% ETc (0.627 kg m^-3^), 80% ETc (0.620 kg m^-3^), and SDI (0.637 kg m^-3^), while it was significantly reduced under 60% ETc (0.551 kg m^-3^) and SF (0.601 kg m^-3^). The reduced BWP by 12.5% under SSDI at 60% ETc compared with 80% ETc was clearly indicative of its poor efficiency (Brar et al., 2021). The SF method revealed least WUE (57%), while values were considerably improved under a SSDI level of 0.8 ETc (80.3%), closely followed by SDI (80.9%), SSDI level of 60% ETc (76.5%), and 100% ETc (78.8%). The better WUE under SDI/SSDI at 0.8 ETc was in conformity with Shruti and Aladakatti (2017), who elucidated higher values under DI applied at 80% ETc.

**Table 8:** Effect of various treatments on bio-physical water productivity, water use efficiency, and nitrogen use efficiency.

Irrigation regimes (IR)							
Nitrogen fertigation schedules (FS)	60% crop evapotranspiration (ETc)	80% ETc	100% ETc	Mean	Control 1		Control 2
Bio-physical water productivity (kg m^-3^)							
75% RDN	0.533	0.602	0.619	0.585			
100% RDN	0.570	0.638	0.636	0.614	0.601	0.637	
Mean	0.551	0.620	0.627				
LSD (*p* = 0.05)	IR = 0.023; FS = 0.018; IR*FS = NS; IR*FS *vs*. controls = 0.017			
Water use efficiency (%)							
75% RDN	75.4	77.1	74.9	75.8			
100% RDN	77.6	83.5	82.7	81.3	57.0	80.9	
Mean	76.5	80.3	78.8				
Nitrogen use efficiency (kg SCY kg^-1^ N)							
76.5	80.3	78.8	37.0	34.1	25.9	29.3	
100% RDN	24.4	30.7	30.9	28.6			
Mean	26.9	33.2	33.9				
LSD (*p* = 0.05)	IR = 1.3; FS = 1.0; IR*FS = NS; IR*FS *vs*. controls = 1.4			

RDN, recommended dose of nitrogen; NS, non-significant; LSD, least significant difference; Control 1, surface flood irrigation and soil application of 100% RDN through urea broadcasting; Control 2, surface drip irrigation at 80% ETc with fertigation of 100% RDN.

Among the fertigation levels, 100% RDN recorded a numerically higher BWP of 0.614 kg m^-3^ over 75% RDN and SF. However, SSDF of 75% and 100% RDN exhibited better WUE over broadcasting fertilizer (Jayakumar et al., 2015). These results clearly established that SSDF of either 80 or 100% ETc along with 100% RDN recorded better BWP and WUE over the SF method despite the limited application of water. Thus, SSDF can become an effective tool for increasing the cotton productivity as well as water use efficiency without sacrificing SCY (Singh et al., 2022). Further details are elaborated in the following subsections.

### Actual crop evapotranspiration

Among the studied treatments, the mean evapotranspiration (Eta) at Faridkot during 2019 was 504.6, 530.2, 553.9, 576.8, 554.6, 580.6, 485.6, and 555.2 mm for T_1_ to T_8_, respectively. During 2020, the respective ETa values were 515.3, 534.7, 572.3, 584.8, 573.4, 582.6, 486.7, and 562.6 mm. At Abohar, the ETa values during 2019 were 380.1, 385.6, 404.6, 451.1, 403.7, 462.8, 345.8, and 410.8 mm for T_1_ to T_8_, respectively. The respective ETa value during 2020 was 517.4, 541.2, 530.5, 585.3, 543.4, 588.8, 544.6, and 590.2 mm. Reduction in ETa at Abohar during 2019 compared with a later year was observed, while at Faridkot the values were akin during both study years (**Tables 6**, **7**). Higher ETa values for treatments receiving more water and fertilizer under drip fertigation resulted in vigorous cotton growth, which might have led to variability in the uptake of water and its distribution. The higher ETa in 2020 might be owing to more rainfall which was primarily accountable for continuous soil wetting, thus leading to a higher evaporative loss (Dar et al., 2017). The variation in ETa over the seasons and locations was owing to the huge variation in the amount of rainfall and its distribution (**Figure 2**), which could have resulted into distinct effects on soil wetting followed by evaporative loss and crop water uptake. The variation among irrigation regimes is primarily owing to the variable quantity of IWA (**Tables 6**, **7**). The higher ETa was observed under T_6_, which received the maximum quantity of irrigation through SSDF, as a linear relationship has been observed between SCY and ETa (**Figure 4**) with the *R* ^2^ value ranging from 0.723 to 0.983 for individual and pooled data over the seasons and locations, respectively. The increase in ETa with IWA is in agreement with Irmak et al. (2016).

### Bio-physical water productivity, water use efficiency, and economic water productivity

The higher BWP was recorded in T_4_ treatment (0.638 kg m^-3^), while the lowest (0.533 kg m^-3^) was observed in T_1_ treatment (**Table 8**). The BWP declined owing to a huge reduction in SCY (**Table 5**) compared with the reduced crop ETa (**Tables 6**, **7**). Among drip irrigation regimes, the BWP values were 0.551, 0.620, and 0.627 kg m^-3^ for 60% ETc, 80% ETc, and 100% ETc, respectively (**Table 8**). The improvement in BWP with any hike in irrigation level could be owing to the reason that IWA just fulfilled the required soil water deficit. This means that treatments which received more irrigation water experienced lesser water stress compared with treatments receiving lesser irrigation. As a result, the SCY was statistically better under 80% ETc and 100% ETc treatments having a high IWA compared with 60% ETc and resulted in higher BWP (Sun et al., 2006; Dar et al., 2017). When averaged over the years, a linear relationship between ETa and SCY was observed, with *R* ^2^ value of 0.723 at Abohar and 0.983 for Faridkot and *R* ^2^ of 0.936 (**Figure 4C**) and *R* ^2^ of 0.891 when averaged over the locations and years. This clearly elucidated that the efficient use of water by the cotton plants increased the SCY. Therefore, irrigating cotton with an adequate amount of water at regular intervals through drip might enhance the SCY and water productivity with additional saving of water compared with surface flood method.

### Comparison of water use efficiency under SSDF treatments vs. surface flood

The WUE in SSDF treatments ranged from 75.4 to 83.5%, compared with SF (57.0%). The highest WUE of 83.5% was recorded in T_4_ treatment, *i*.*e*., drip irrigation at 80% ETc along with 100% RDN (**Table 8**), which elucidated that a huge water can be saved by adopting novel water-savvy techniques. During 2019, applied water under SF has been 56% and 110% higher compared with what was applied in SSDI at 100% ETc for Faridkot and Abohar, respectively. The corresponding values were higher by 24.6% and 42.5% during 2020, which was indicative of huge water saving.

### Nitrogen use efficiency

Nitrogen is one of the most important limiting factors governing the growth and productivity of crop plants. The present findings clearly established a reduction in the NUE with each increase in N level. Within irrigation regimes, SSDI of 100% ETc recorded a higher NUE (33.9 kg SCY kg^-1^ N) which was closely followed by 80% ETc (33.2 kg SCY kg^-1^ N), while 60% ETc resulted in a significantly lowest value (26.9 kg SCY kg^-1^ N). Furthermore, NUE under SF (Control 1) has been 3.8%, 28.1%, and 30.8% lesser compared with SSDI of 60% ETc, 80% ETc, and 100% ETc, respectively (**Table 8**).

Among fertigation levels, SSDF of 75% RDN (34.1 kg SCY kg^-1^ N) recorded a statistically better NUE by 19.2% over 100% RDN (28.6 kg SCY kg^-1^ N), which was in agreement with Singh et al. (2018) who reported 27% higher NUE under 75% RDN over 100% RDN. However, both SSDF levels of 75% and 100% RDN exhibited increased NUE by 31.6% and 10.4%, respectively, over SF (25.9 kg SCY kg^-1^ N). The higher NUE under SSDF has been primarily due to the application of nitrogen directly to the root zone, which, in turn, minimized volatilization and leaching losses and consequently improved the yield (Brar et al., 2022). Moreover, the application of N in 10 equal splits under SSDF might have checked the N losses in the soil compared with only two splits under the broadcasting method, which further helped to enhance NUE, in agreement with Bharathraj et al. (2015) wherein 47% higher efficiency has been observed under fertigation compared with soil application.

### Fiber quality parameters

Ginning turnout was significantly reduced under SF (31.9%) compared with all SDI/SSDF treatments (**Table 9**). However, fiber strength was significantly higher (30.5) under a SSDF combination of 80% ETc along with 100% of RDN, which was in conformity with Zhang et al. (2019). However, fertigation treatments could not differentiate much for either of the studied quality parameters (Magare et al., 2018; Zahid et al., 2021).

**Table 9:** Effect of various treatments on fiber quality parameters.

Irrigation regimes (IR)							
Nitrogen fertigation schedules (FS)	60% crop evapotranspiration (ETc)	80% ETc	100% ETc	Mean	Control 1		Control 2
Ginning turnout (%)							
75% RDN	33.2	33.3	33.4	33.3	31.9	33.2	
100% RDN	33.1	33.8	33.9	33.6			
Mean	33.1	33.5	33.7				
LSD (*p* = 0.05) IR = NS; FS = NS; IR*FS = NS; IR*FS *vs*. controls = 0.99							
Halo length (mm)							
75% RDN	26.4	27.6	27.1	27.0	26.7	26.8	
100% RDN	26.6	26.9	27.1	26.9			
Mean	26.5	27.3	27.1				
LSD (*p* = 0.05) IR = NS; FS = NS; IR*FS = NS; IR*FS *vs*. controls = NS							
Uniformity index							
75% RDN	80.3	80.2	80.4	80.3	80.6	80.7	
100% RDN	80.6	80.7	80.5	80.6			
Mean	80.4	80.5	80.4				
LSD (*p* = 0.05) IR = NS; FS = NS; IR*FS = NS; IR*FS *vs*. controls = NS							
Fiber strength (g tex^-1^)							
75% RDN	29.9	30.8	30.3	30.3	29.5	30.1	
100% RDN	30.1	30.2	29.8	30.0			
Mean	30.0	30.5	30.0				
LSD (*p* = 0.05) IR = 0.44; FS = NS; IR*FS = NS; IR*FS *vs*. controls = 0.47							
Micronaire							
75% RDN	4.20	4.23	4.21	4.21	4.21	4.23	
100% RDN	4.21	4.25	4.20	4.22			
Mean	4.21	4.24	4.20				
LSD (*p* = 0.05) IR = NS; FS = NS; IR*FS = NS; IR*FS *vs*. controls = NS							

### Monetary evaluation

A higher gross return ($2,572.9 ha^-1^), net return ($1,648.9 ha^-1^), and B:C ratio (1.78) have been recorded in the treatment receiving SSDF at 100% ETc with fertigation of 100% RDN. However, SSDI of 80% ETc with 100% RDN recorded much lesser net returns by $12.0 ha^-1^, but with an additional saving of 20% irrigation water (**Table 10**). A statistically lowest gross return ($1,842.6 ha^-1^), net return ($932.0 ha^-1^), and B:C (1.02) was observed under SSDI at 0.6 ETc with 75% RDN, thus making it the least remunerative among the drip combinations studied. Nevertheless, SSDI at 0.8 ETc with 100% RDN recorded significantly higher net returns by 54.1% over the SF method. These findings get fair support from Aladakatti et al. (2012) and Pawar et al. (2015). Reduced B:C under the conventional practice of the SF method (1.11) further substantiated that fertilizer and water application through SSDF are more rewarding (Neelakanth et al., 2019).

**Table 10:** Effect of various treatments on monetary parameters.

Nitrogen fertigation schedules (FS)	Irrigation regimes (IR)								
	60% ETc	80% ETc		100% ETc		Mean	Control 1		Control 2
Cost of cultivation ($ ha^-1^)									
75% RDN	910.5	914.8		919.1		914.8	956.7	919.8	
100% RDN	915.4	919.7		924.0		919.7			
Mean	913.0	917.2		921.5					
Gross returns ($ ha^-1^)									
75% RDN	1842.6	2237.7		2318.4		2132.9	2018.7	2442.0	
100% RDN	2032.7	2556.7		2572.9		2387.4			
Mean	1937.6	2397.2		2445.6					
Net Returns ($ ha^-1^)									
75% RDN	932.0	1322.9		1399.3		1218.1	1062.0	1522.2	
100% RDN	1117.2	1636.9		1648.9		1467.7			
Mean	1024.6	1479.9		1524.1					
Benefit : Cost ratio									
75% RDN	1.02	1.44		1.52		1.33	1.11	1.65	
100% RDN	1.22	1.77		1.78		1.59			
Mean	1.12	1.61		1.65					
Economic water productivity ($ m^-3^)									
75% RDN	0.201	0.265		0.277		0.248			
100% RDN	0.234	0.302		0.302		0.279	0.233	0.294	
Mean	0.218	0.284		0.289					

Economic water productivity remained higher under the SSDI level of 100% ETc and SDI, closely followed by SSDI of 80% ETc, while the least values were exhibited under 60% ETc ($0.218 m^-3^) and the SF method ($0.233 m^-3^). EWP under SF was lower by 17.9% and 20%, respectively, over the SSDI levels of 80% ETc and 100% ETc, which indicated it to be inferior compared with other treatments. Among fertigation levels, SSDF of 100% RDN recorded better EWP ($0.279 m^-3^) over 75% RDN. However, both SSDF levels of 75% and 100% RDN exhibited increased EWP by 7% and 17%, respectively, over Control 1. The SSDF of 80% ETc with 100% RDN and 100% ETc with 100% RDN recorded 29.6% higher EWP over SF. These findings clearly elucidated the advantage of SSDF in increasing monetary advantage over SF.

## Conclusion

The available freshwater for agrarian purposes in northwestern India is continuously declining due to reduced river flows, leading to sub-optimal canal water supply and changed levels of precipitation. Hence, effective irrigation strategies may help save water without sacrificing the crop productivity. Here we elucidated for the first time that, in northwestern India, the SSDF technique may result into significant savings of irrigation water due to lesser drainage loss compared with SF and is a potentially viable option for cotton cultivation. The highest WUE (83.5%) under SSDF of 80% ETc and 100% RDN, along with improvised BWP (0.638 kg m^-3^) and better SCY (3,455 kg ha^-1^), established it to be most efficient among the treatments tested. Furthermore, it also resulted in 26.6% higher SCY, 6.1% better BWP, and 29.6% higher EWP along with 18.5% higher NUE than surface flood. Therefore, implementation of SSDF in cotton would not only save a large quantity of irrigation water but also support more areas under micro-irrigation in sustaining a better yield. Therefore, growing cotton with optimized sub-surface drip fertigation would be an efficient and economically viable water-savvy strategy in northwestern India.

## Data availability statement

The original contributions presented in the study are included in the article/supplementary material. Further inquiries can be directed to the corresponding authors.

## Author contributions

Conceptualization: KS. Methodology: KS, PS, and MS. Investigation: KS, PS, MS, and SM. Resources: KS, MS, and AS. Writing of the original draft: KS, PS, MS, and SM. Editing: KS, RI, IA-A, MH-u-R, and AS. All authors contributed to the article and approved the submitted version.

## References

[B1] Aladakatti Y. R. Hallikeri S. S. Nand agavi R. A. Shivamurthy D. Malik R. (2012). “Precision irrigation and fertigation to enhance the productivity and economic returns of bt cotton in vertisols,” in Proc 3rd agro-informat precision agriculture(Hyderabad, India: Agro-Informatics and Precision Agriculture), 341–343.

[B2] Ali H. Arooj M. Sarwar N. Areeb A. Shahzad A. N. Hussain S. (2017). Sustainable weed management strategy in cotton crop. Planta Daninha 35, 1–12. doi: 10.1590/S0100-83582017350100052

[B3] Anonymous (2021). All India coordinated research project on cotton, project co-ordinator. (PC) report., (2021) (Nagpur, India: CICR). Available at: https://www.cicr.org.in/aicrp-2021/2_PC_Report.pdf.

[B4] Anonymous (2022). Cotton package of practices for crops of punjab - kharif, (2022) (Ludhiana, India: Punjab Agricultural University), Pp:37–Pp:51. Available at: https://www.pau.edu/content/ccil/pf/pp_kharif.pdf.

[B5] Ayyadurai P. Manickasundaram P. (2014). Growth, nutrient uptake and seed cotton yield as influenced by foliar nutrition and drip fertigation in cotton hybrid. Int. J. Agric. Sci. 10 (1), 276–279. doi: 10.20546/ijcmas.2017.609.366

[B6] Ben-Gal A. Lazorovitch N. Shani U. (2004). Subsurface drip irrigation in gravel filled cavities. Vadose Zone J3. 4), 1407–1413. doi: 10.2136/vzj2004.1407

[B7] Bharathraj H. R. Joshi M. Vishaka G. V. (2015). Effect of surface fertigation on nutrient uptake, fertilizer use efficiency and economics of inter-specific hybrid *Bt* cotton. Univ. J. Agric. Res. 3, 46–48. doi: 10.13189/ujar.2015.030202

[B8] Black G. R. Hartage K. H. (1986). Bulk density. in: Klute, a. (ed), methods of soil analysis. part i. physical and mineralogical methods. Amer. Soc Agron. Soil Sci. Madison WI pp, 363–375. doi: 10.2136/sssabookser5.1.2ed.c13

[B9] Brar A. S. Buttar G. S. Singh M. Singh S. Vashist K. K. (2021). Improving bio−physical and economic water productivity of menthol mint (*Mentha arvensis* l.) through drip fertigation. Irrig. Sci 39 (4), 505–516. doi: 10.1007/s00271-021-00722-6

[B10] Brar A. S. Kaur K. Sindhu V. K. Tsolakis N. Srai J.S. (2022). Sustainable water use through multiple cropping systems and precision irrigation. J. Cleaner Prod. 333, 130117. doi: 10.1016/Jjclepro.2021.130117

[B11] Cetin O. Kara A. (2019). Assessment of water productivity using different drip irrigation systems for cotton. Agric. Water Manage. 223, 1–9. doi: 10.1016/j.agwat.2019.105693

[B12] Dar E. A. Brar A. S. Singh K. B. (2017). Water use and productivity of drip irrigated wheat under variable climatic and soil moisture regimes in north-West, India. Agric. Ecosyst. Environ. 248, 9–19. doi: 10.1016/Jagee.2017.07.019

[B13] FernÃ¡ndez J. E. Alcon F. Diaz-Espejo A. Hernandez-Santana V. Cuevas M. V. (2020). Water use indicators and economic analysis for on-farm irrigation decision: A case study of a super high density olive tree orchard. Agric. Water Manage. 237, 106074. doi: 10.1016/Jagwat.2020.106074

[B14] Gondal M. R. Saleem M. Y. Rizvi S. A. Riaz A. Naseem W. Muhammad G.. (2021). Assessment of drought tolerance in various cotton genotypes under simulated osmotic settings. Asian J. Agric. Biol. 2, 1–10. doi: 10.35495/ajab.2020.08.437

[B15] Harish J. Rajkumar B. Pawar D. D. Kale K. D. (2017). Nutrient availability in *Bt*cotton by using drip fertigation under different phosphorous sources. Int. J. Res. Sci. Tech. 6, 406–410. doi: 10.17577/IJERTV6IS060233

[B16] Hashem M. S. El-Abedin T. Z. Al-Ghobari H. M. (2018). Assessing effects of deficit irrigation techniques on water productivity of tomato for subsurface drip irrigation system. *Int. j.* agric. Biol. Eng. 11 (4), 156–167. doi: 10.25165/j.ijabe.20181104.3846

[B17] Ibragimov N. Evet S. R. Esanbekov Y. Kamilov B. Mirzaev L. Lamers J. P. A. (2007). Water use efficiency of irrigated cotton in Uzbekistan under drip and furrow irrigation. Agric. Water Manage. 90, 112–120. doi: 10.1016/j.agwat.2007.01.016

[B18] Imran M. Ali A. Safdar M. E. (2021). The impact of different levels of nitrogen fertilizer on maize hybrids performance under two different environments. Asian J. Agric. Biol. 4, 1–10. doi: 10.35495/ajab.2020.10.527

[B19] Ines A. V. M. Honda K. Das Gupta A. Droogers P. Clemente R. S. (2006). Combining remote sensing-simulation modeling and genetic algorithm optimization to explore water management options in irrigated agriculture. Agric. Water Manage. 83, 221–232. doi: 10.1016/j.agwat.2005.12.006

[B20] Irmak S. Djaman. K. Rudnick D. R. (2016). Effect of full and limited irrigation amount and frequency on subsurface drip-irrigated maize evapotranspiration, yield, water use efficiency and yield response factors. Irrig. Sci. 34, 271–286. doi: 10.1007/s00271-016-0502-z

[B21] Jackson M. L. (1967). Soil chemical analysis. prentice hall of India, private limited (New Delhi: Prentice Hall of India, Private Limited, New Delh). doi: 10.1002/jpln.19590850311

[B22] Janat. (2008). Response of cotton to irrigation methods and nitrogen fertilization: yield components, water use efficiency, nitrogen uptake and recovery. Commun. Soil Sci. Plant Anal. 39, 2282–2302. doi: 10.1080/00103620802292293

[B23] Jayakumar M. Surendran U. Manicksundram P. (2015). Drip fertigation program on growth, crop productivity, water and fertilizer use efficiency of *Bt*cotton in semi arid tropical region of India. Commun. Soil Sci. Plant Anal. 46, 293–300. doi: 10.1080/00103624.2014.969403

[B24] Kaur A. Brar A. S. (2016). Influence of mulching and irrigation scheduling on productivity and water use of turmeric in north western India. Irrig. Sci. 34, 261–269. doi: 10.1007/s00271-016-0501-0

[B25] Kumar D. S. Sharma R. Brar A. S. (2021). Optimising drip irrigation and fertigation schedules for higher crop and water productivity of oilseed rape (*Brassica napus* l.). Irrig. Sci 39 (4), 535–548. doi: 10.1007/s00271-020-00714-y

[B26] Magare P. N. Katkar R. N. Jadhao S. D. (2018). Effect of fertigation on yield, quality and soil fertility status under cotton grown in vertisol. Int. J. Chem. Stud. 6 (2), 42–46.

[B27] Martinez J. Reca J. (2014). Water use efficiency of surface drip irrigation versus an alternative sub-surface drip irrigation method. J. Irrig. Drain. Eng. 140, 1–9. doi: 10.1061/(ASCE)IR.1943-4774.0000745

[B28] Mchugh A. D. Bhattarai S. Midmore D. J. (2008). Effects of sub-surface drip irrigation rates and furrow irrigation for cotton grown on a vertisol on off-site movements of sediments, nutrients and pesticides. *Agric* . Sustain. Dev. 28, 507–519. doi: 10.1051/agro:2008034

[B29] Merwin H. D. Peech M. (1950). Exchangeability of soil potassium in sand, silt and clay fractions as influenced by the nature of complementary exchangeable cations. Proc. Soil America 15, 125–128. doi: 10.2136/sssaj1951.036159950015000C0026x

[B30] Mishra S. K. Kaur V. Singh K. (2021). Evaluation of DSSAT-CROPGRO-cotton model to simulate phenology, growth and seed cotton yield in north-western India. Agron. J. 113, 3975–3990. doi: 10.1002/agj2.20788

[B31] Neelakanth J. K. Rajkumar S. Gundlur S. S. Dasar G. V. (2019). Effect of surface and sub-surface drip irrigation system on seed cotton in *vertisols*of malaprabha command in northern karnataka. J. Pharmacogn. Phytochem. 8 (2), 956–958.

[B32] Olsen S. R. Cole C. V. Waternade F. S. Dean L. A. (1954). Estimation of available phosphorous in soil by extraction with sodium bicarbonate. USDA Circ. 939, 1–19.

[B33] Patil N. G. Ramamurthy V. Venugopalan M. V. Challa O. (2009). Effect of drip irrigation on productivity and water-use efficiency of hybrid cotton. (*Gossypium hirsutum*) in typic haplusterts. Ind. J. Agric. Sci. 79, 118–121.

[B34] Pawar N. Bishnoi. D. K. Singh M. Dhillon A. (2015). Comparative economic analysis of drip irrigation vis-a-vis flood irrigation system on productivity of *Bt* cotton in haryana. *Agric* . Sci. Digest. 35 (4), 300–303. doi: 10.18805/asd.v35i4.6863

[B35] Pereira L. S. Cordery I. Lacovides I. (2012). Improved indicators of water use performance and productivity for sustainable water conservation and saving. Agric. Water Manage. 108, 39–51. doi: 10.1016/j.agwat.2011.08.022

[B36] Perry C. Pasquale S. Allen R. G. Burt C. M. (2009). Increasing productivity in irrigated agriculture: agronomic constraints and hydrological realities. Agric. Water Manage. 96, 1517–1524. doi: 10.1016/j.agwat.2009.05.005

[B37] Perry C. Pasquale S. Karajeh F. Food and Agriculture Organization of the United Nations (2017). “Does improved irrigation technology save water,” in Discussion paper on irrigation and sustainable water resources management in the near East and north Africa(Cairo: Food and Agriculture Organization of the United Nations, Cairo). Available at: https://www.fao.org/3/I7090EN/i7090en.pdf.

[B38] Piper C. S. (1966). Soil and plant analysis (New York: International Science Publisher). doi: 10.1002/jps.3030350611

[B39] Prajapati G. V. Subbaiah R. (2018). Combined response of irrigation system regimes and mulching on productivity of *Bt* cotton. J. Agrometeorol. 20, 47–51.

[B40] Rahim H. Mian I. A. Muhammad A. Ahmad S. Khan Z. (2020). Soil fertility status as influenced by the carryover effect of biochar and summer legumes. Asian J. Agric.and Biol. 8 (1), 11–16. doi: 10.35495/ajab.2019.05.198

[B41] Richards L. A. Weaver L. R. (1943). Fifteen-atmosphere percentage as related to the permanent wilting percentage. Soil Sci 56, 331–39. doi: 10.1097/00010694-194311000-00002

[B42] Roopashree M. Rajkumara S. Neelakanth J. K. (2016). Effect of surface and sub-surface drip irrigation at different ETc levels on growth and yield of *Bt* cotton. (*Gossypium hirsutum*L.). J. Farm Sci. 29, 456–460.

[B43] Sahoo P. Brar A. S. Sharma S. (2018). Effect of methods of irrigation and sulphur nutrition on seed yield, economic and bio-physical water productivity of two sunflower (*Helianthus annus* l.) hybrids. Agric. Water Manage. 206, 158–164. doi: 10.1016/j.agwat.2018.05.009

[B44] Sampathkumar T. Krishnasamy S. Ramesh S. Prabukumar G. Gobi R. (2006). Growth, nutrient uptake and seed cotton yield of summer cotton as influenced by drip, surface irrigation methods and mulching practices. *Res. j. agric* . Biol. Sci. 2, 420–422.

[B45] Shruti M. Y. Aladakatti Y. R. (2017). Effect of drip irrigation and fertigation on yield, economics and water use efficiency of intra-*hirsutumBt* cotton. J. Farm Sci. 30, 185–189.

[B46] Sidhu H. S. Jat M. L. Singh Y. Sidhu R. K. Gupta N. Singh P.. (2019). Sub-Surface drip fertigation with conservation agriculture in a rice-wheat system: A breakthrough for addressing water and nitrogen use efficiency. Agric. Water Manage. 216, 273–283. doi: 10.1016/j.agwat.2019.02.019

[B47] Singh M. Bhati A. S. (2018). Nutrient use in cotton grown under drip irrigation system innorth-western India. J. Crop Weed. 14, 122–129.

[B48] Singh K. Brar A. S. Singh H. P. (2018). Drip fertigation improves water and nitrogen use efficiency of *Bt* cotton. J. Soil Water Cons. 73, 549–557. doi: 10.2489/jswc.73.5.549

[B49] Singh K. Mishra S. K. Singh M. Singh K. Brar A. S. (2022). Water footprint assessment of surface and subsurface drip fertigated cotton-wheat cropping system – a case study under semi-arid environments of Indian punjab. J. Cleaner Prod. 365, 132735. doi: 10.1016/Jjclepro.2022.132735

[B50] Singh K. Singh H. P. Mishra S. K. (2020). Irrigation module and sowing dates affect seed cotton yield, quality, productivity indices and economics of cotton in north-western india. *Commun. soil sci* . Plant Annal. 51 (7), 919–931. doi: 10.1080/00103624.2020.1744633

[B51] Sinha I. Buttar G. S. Brar A. S. (2017). Drip irrigation and fertigation improve economics, water and energy productivity of spring sunflower (*Helianthus annuus* L.) in Indian Punjab. Agric. Water Manage 185, 58–64. doi: 10.1016/j.agwat.2017.02.008

[B52] Subbiah B. V. Asija G. L. (1956). A rapid procedure for the estimation of available nitrogen in soils. Curr. Sci. 25, 259–260.

[B53] Sun H. Y. Liu C. M. Zhang X. Y. Shen Y. J. Zhang Y. Q. (2006). Effects of irrigation on water balance, yield and WUE of winter wheat in the north China plain. Agric. Water Manage. 85, 211–218. doi: 10.1016/j.agwat.2006.04.008

[B54] Thanappan S. Hosamani S. R. Chandrappa M. N. (2020). Rill treatments to enhance nutrient rich soil, a case study. Asian J. Agric. Biol. 8 (2), 186–193. doi: 10.35495/ajab.2019.06.242

[B55] Walkley A. Black C. A. (1934). An examination of the digtjareff method for determination of soil organic matter and a proposed modification of chromic acid titration method. Soil Sci. 37, 29–38. doi: 10.1097/00010694-193401000-00003

[B56] Yadav B. S. Chauhan R. P. S. (2016). Drip fertigation technology for enhancing water and nutrient use efficiency in arid agro-ecosystem of irrigated northwestern rajasthan. Ann. Arid Zone. 55, 139–145.

[B57] Zahid N. Ahmed M. J. Tahir M. M. Maqbool M. Shah S. Z. A. Hussain S. J.. (2021). Integrated effect of urea and poultry manure on growth, yield and postharvest quality of cucumber (*Cucumis sativus* l.). Asian J. Agric. Biol. 1, 1–9. doi: 10.35495/ajab.2020.07.381

[B58] Zhang H. Liu H. Wang S. Guo X. Ge L. Sun J. (2019). Variations in growth, water consumption and economic benefit of transplanted cotton after winter wheat harvest subjected to different irrigation methods. Sci. Rep. 9, 1–11. doi: 10.1038/s41598-019-51391-7 31628374 PMC6802123

