# Corrigendum: Sub-surface drip fertigation improves seed cotton yield and monetary returns

**DOI:** 10.3389/fpls.2023.1139681

**Published:** 2023-02-02

**Authors:** Kulvir Singh, Prabhsimran Singh, Manpreet Singh, Sudhir Kumar Mishra, Rashid Iqbal, Ibrahim Al-Ashkar, Muhammad Habib-ur-Rahman, Ayman El Sabagh

**Affiliations:** ^1^ Regional Research Station, Punjab Agricultural University, Faridkot, Punjab, India; ^2^ Department of Agronomy, Faculty of Agriculture and Environment, The Islamia University of Bahawalpur, Bahawalpur, Pakistan; ^3^ Department of Agronomy, Faculty of Agriculture and Environment, The Islamia University of Bahawalpur Pakistan, Charbagh, India; ^4^ Department of Plant Production, College of Food and Agriculture, King Saud University, Riyadh, Saudi Arabia; ^5^ Crop Science, Institute of Crop Science and Resource Conservation (INRES), University of Bonn, Bonn, Germany; ^6^ Department of Agronomy, Faculty of Agriculture, Kafrelsheikh University, Kafrelsheikh, Egypt

**Keywords:** bio-physical water productivity, drip fertigation, economic water productivity, nitrogen use efficiency, surface flood method, water use efficiency

In the published article, there was an error in affiliation 3. Instead of “Center for Plant Science and Biodiversity, University of Swat, Charbagh, Pakistan”, it should be “Department of Agronomy, Faculty of Agriculture and Environment, The Islamia University of Bahawalpur, Bahawalpur, Pakistan”.

An author name was incorrectly written as “Rana Rashid Iqbal”. The correct spelling is “Rashid Iqbal”.

The authors apologize for these errors and state that this does not change the scientific conclusions of the article in any way. The original article has been updated.

